# Efficient NH_3_-based process to remove chlorine from electron beam deposited ruthenium produced from (η^3^-C_3_H_5_)Ru(CO)_3_Cl

**DOI:** 10.1038/s41598-020-67803-y

**Published:** 2020-07-02

**Authors:** Markus Rohdenburg, Hannah Boeckers, Christopher R. Brewer, Lisa McElwee-White, Petra Swiderek

**Affiliations:** 1 Institute for Applied and Physical Chemistry (IAPC), Fachbereich 2 (Chemie/Biologie), University of Bremen, Leobener Str. 5 (NW2), 28359 Bremen, Germany; 2 Department of Chemistry, University of Florida, Gainesville, FL 32611-7200 USA

**Keywords:** Chemistry, Nanoscience and technology

## Abstract

The fabrication of Ru nanostructures by focused electron beam induced deposition (FEBID) requires suitable precursor molecules and processes to obtain the pure metal. So far this is problematic because established organometallic Ru precursors contain large organic ligands, such as cyclopentadienyl anions, that tend to become embedded in the deposit during the FEBID process. Recently, (η^3^-C_3_H_5_)Ru(CO)_3_X (X = Cl, Br) has been proposed as an alternative precursor because CO can easily desorb under electron exposure. However, allyl and Cl ligands remain behind after electron irradiation and the removal of the halide requires extensive electron exposures. Auger electron spectroscopy is applied to demonstrate a postdeposition purification process in which NH_3_ is used as a reactant that enhances the removal of Cl from deposits formed by electron irradiation of thin condensed layers of (η^3^-C_3_H_5_)Ru(CO)_3_Cl. The loss of CO from the precursor during electron-induced decomposition enables a reaction between NH_3_ and the Cl ligands that produces HCl. The combined use of electron-stimulated desorption experiments and thermal desorption spectrometry further reveals that thermal reactions contribute to the loss of CO in the FEBID process but remove only minor amounts of the allyl and Cl ligands.

## Introduction

Focused electron beam induced deposition (FEBID) is a state-of-the art nanofabrication process, capable of producing freestanding nanometer-sized structures with arbitrary shape^1–4^. In FEBID, precursor molecules adsorbed on a surface are decomposed under a tightly focused high-energy electron beam to produce the desired solid material. Deposition of a particular metal thus requires a suitable organometallic precursor. Ideally, this precursor should decompose completely upon electron irradiation to leave only the metal behind, while the ligands are efficiently converted to volatile byproducts that desorb from the deposit. However, metallic nanostructures produced by FEBID are often contaminated by considerable amounts of unwanted elements from incomplete ligand desorption, so that the targeted properties are not achieved^1,5–7^. Therefore, many common FEBID precursors require strategies to purify the deposit^5^.

A wide variety of purification strategies for FEBID has been reported, including thermal reactions, prolonged electron exposure, process gases, or a combination of at least two of these approaches^5^. A recent successful development is to use H_2_O as process gas^8,9^. For instance, carbon-rich deposits produced from trimethyl(methylcyclopentadienyl)platinum(IV) (MeCpPtMe_3_) were converted to pure Pt by post-deposition electron irradiation in the presence of H_2_O^8^. As another example, a FEBID process that simultaneously dosed dimethyl(acetylacetonato)gold(III) (AuMe_2_(acac)) and H_2_O yielded a deposit with Au content above 80%, in contrast to low-purity deposits produced from AuMe_2_(acac) alone^9^. A study of such processes in the case of MeCpPtMe_3_ by surface science techniques showed that H_2_O converts the carbon content to CO and CH_4_^10^.

An important application of FEBID is the repair of masks for photolithography^11,12^. In this area, the emerging nanofabrication tool extreme ultraviolet lithography (EUVL), requires capping layers that protect the masks during processing and exposure. Ru has been identified as a material of choice for this purpose^11^. Therefore, FEBID processes that yield high purity Ru are of interest for EUVL mask repair. However, the best commonly used precursor for Ru, bis(ethylcyclopentadienyl)ruthenium(II) ((EtCp)_2_Ru) retains large amounts of carbon during its electron-induced decomposition, while removal of carbon from the deposit by electron irradiation in the presence of O_2_ results in oxidation of the metal^11^. Novel precursors for FEBID processes aiming at pure Ru deposits are of interest to address these challenges. Therefore, a class of Ru precursors, namely η^3^-allyl ruthenium tricarbonyl halides ((η^3^-C_3_H_5_)Ru(CO)_3_X, X = Cl, Br), has been proposed and its electron-induced chemistry has been studied in the gas-phase^13^ and on surfaces^14^. Also, the performance of (η^3^-C_3_H_5_)Ru(CO)_3_Br in the FEBID process was investigated^15^. The advantage of these precursors lies in the lower number of carbon atoms as compared to (EtCp)_2_Ru. Additionally, the CO ligands, which contribute half of the carbon content in the precursor, are particularly facile to remove. However, extensive electron irradiation is required to remove the halide ligand^14^.

**Figure 1:**
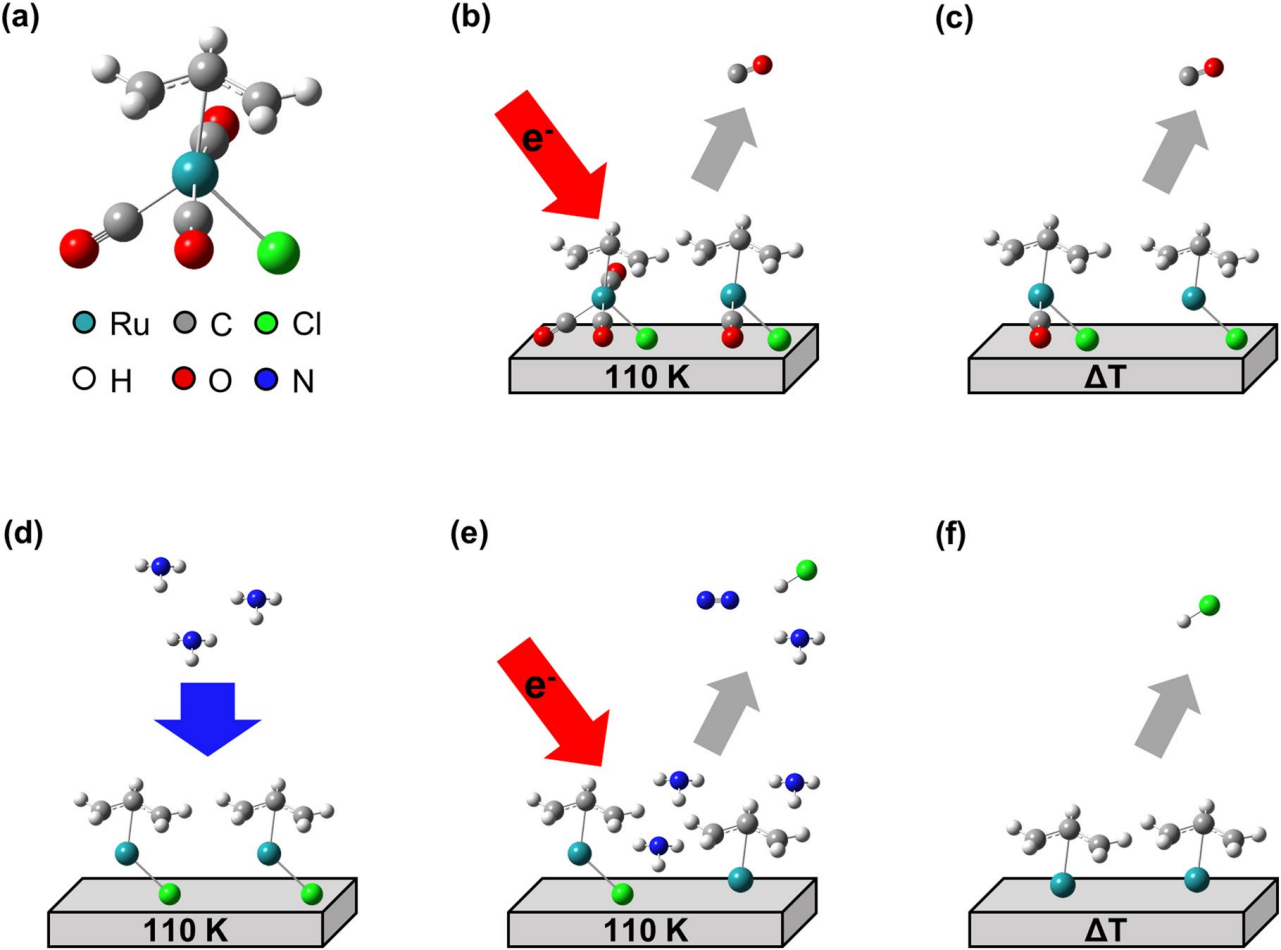
Overview of experiments to unravel the chemistry that underlies the electron-induced formation of deposits from the Ru precursor (η^3^-C_3_H_5_)Ru(CO)_3_Cl and the NH_3_-assisted purification process aiming at removal of Cl from the deposits. **(a)** Structure of the precursor and colour scheme of the relevant elements. Deposits were produced by **(b)** electron-stimulated desorption (ESD) from thin condensed layers of (η^3^-C_3_H_5_)Ru(CO)_3_Cl on Ta held at 110 K and **(c)** subsequent thermal desorption spectrometry (TDS) during which the sample was annealed to 450 K at a rate of 1 K/s. The deposit was used in the subsequent purification experiments consisting of several cycles in each of which **(d)** NH_3_ was condensed onto the deposit at 110 K, followed by **(e)** an ESD and **(f)** a TDS experiment. Desorption of volatile species was monitored in all experiments by mass spectrometry (MS).

We have recently studied the electron-induced decomposition of cisplatin (*cis*-Pt(NH_3_)_2_Cl_2_) and shown that its NH_3_ ligands assist with the removal of Cl from the resulting deposit^16,17^. Under electron exposure, the NH_3_ ligands decompose to deliver hydrogen that converts the Cl ligands to volatile HCl^16^. Herein, we show that this reducing action of NH_3_ can also be exploited to purify deposits produced from thin condensed layers of (η^3^-C_3_H_5_)Ru(CO)_3_Cl (Fig. 1a) by electron irradiation at cryogenic temperature (Fig. 1b) and subsequent annealing (Fig. 1c). The chemistry underlying the purification process was studied by repeatedly condensing NH_3_ on the cold deposit (Fig. 1d) followed by electron irradiation (Fig. 1e) and a further annealing step (Fig. 1f). During irradiation sequences, the electron-stimulated desorption (ESD) of volatile products was monitored. Thermal desorption spectrometry (TDS) performed during the annealing steps revealed not only products that were formed during electron irradiation and desorb at higher temperature, but also products resulting from thermal reactions that set in above specific temperatures. Furthermore, reflection absorption infrared spectroscopy (RAIRS) monitored the remaining CO ligands after electron exposure and annealing. As introduced recently^10^, the combination of ESD and TDS allows us to identify contributions of thermal reactions to the electron-induced chemistry involved in FEBID. This bridges the gap between the previous surface science studies^14^ and the actual FEBID experiments^15^ on (η^3^-C_3_H_5_)Ru(CO)_3_X precursors. Using Auger electron spectroscopy (AES), we then provide evidence that NH_3_ is in fact a suitable process reagent to remove Cl, and potentially also other halides during FEBID processes using halide containing precursors.

## Results

### Electron-stimulated desorption from adsorbed (η^3^-C_3_H_5_)Ru(CO)_3_Cl

**Figure 2:**
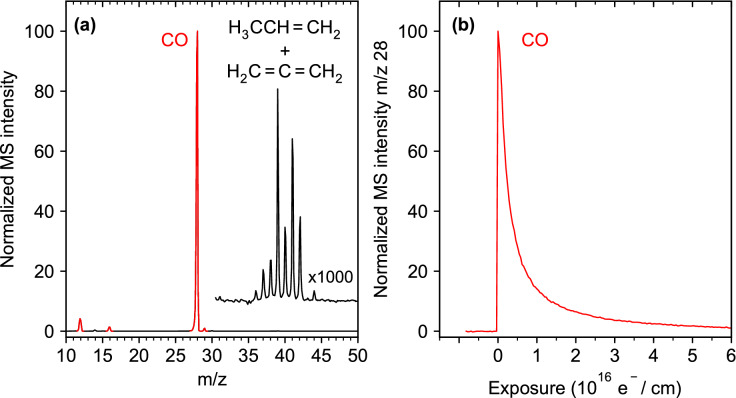
Electron-stimulated desorption (ESD) data obtained during electron exposure at *E*_0_ = 31 eV from an adsorbed layer of (η^3^-C_3_H_5_)Ru(CO)_3_Cl on Ta held at 110 K. **(a)** Mass spectrum recorded during the initial stage of electron exposure and blow-up of the spectrum above *m/z* 30. **(b)** Decay of CO signal (*m/z* 28) as function of electron exposure. The total exposure of this experiment amounted to 1.25·10^17^ e^−^/cm^2^ and the sample current dropped slightly from *I*_p_ = 100 μA to 96 μA during the entire irradiation period.

The electron-induced degradation of thin (η^3^-C_3_H_5_)Ru(CO)_3_Cl layers on Ta was studied at 110 K by monitoring ESD of volatile products as visualized in Fig. 1b. A representative data set is shown in Fig. 2. In line with previous results^14^, only CO was detected with noticeable intensity during irradiation, as indicated by the signals at *m/z* 28, *m/z* 16 and *m/z* 12 in the mass spectrum (Fig. 2a). Very small signals in the range *m/z* 36–42 become visible when the spectrum is blown up by a factor of 1,000 (range above *m/z* 30 in Fig. 2a), which is characteristic of hydrocarbon compounds with three carbon atoms (C_3_ hydrocarbons), such the allyl ligands of (η^3^-C_3_H_5_)Ru(CO)_3_Cl. Our ESD experiments detect neutral species for which the desorption cross sections are at least an order of magnitude higher than for ions^18^, with the majority of products being stable neutral species^10,16^. However, the intensity pattern observed here matches neither the mass spectrum of propene (H_2_C = CH-CH_3_) nor that of propadiene (H_2_C = C = CH_2_) (compare mass spectra in^19^). Propene and propadiene would both derive from H-abstraction processes involving an allyl radical. In the case of propene, C1 or C3 of the allyl radical abstracts H from another species. Propadiene would arise from loss of the C2 hydrogen from an allyl radical. However, by overlaying both mass spectra weighted by suitable factors, the mass spectrum observed during ESD is roughly reproduced (Supplementary Information, Fig. S1), suggesting that H transfer occurs as a minor reaction channel. Note that Cl (*m/z* 35) is absent from the mass spectrum acquired during electron exposure.

The *m/z* 28 signal decayed during irradiation and nearly ceased after an electron exposure of 6·10^16^ e^-^/cm^2^ (Fig. 2b). We note that ESD of CO during the present experiments at 31 eV decayed on a similar time scale as previously observed for a layer of the related compound, (η^3^-C_3_H_5_)Ru(CO)_3_Br, on Au with a similar thickness (stated as 1–2 nm), but irradiated at 500 eV^14^. This is surprising regarding previous results for cisplatin which decomposed much more rapidly at 500 eV than at 50 eV within the same electron exposure^16^. Notably, the different halogen does not appear to affect the decomposition rate of the Ru precursors because the loss of CO proceeded at a similar rate for (η^3^-C_3_H_5_)Ru(CO)_3_Br and (η^3^-C_3_H_5_)Ru(CO)_3_Cl according to XPS^14^. We can also exclude charging of the samples in our experiment as origin of the rapid decay of ESD because the sample current was nearly constant during the entire irradiation period. Additionally, an effect of the different underlying metal is unlikely because the secondary electron (SE) yields are similar for Ta and Au^20^, so that it is not conceivable that more SEs should be produced at 31 eV from Ta in the present experiment than at 500 eV from Au as used previously^14^. Therefore, the rapid decomposition of (η^3^-C_3_H_5_)Ru(CO)_3_Cl at 31 eV must be an inherent property of the precursor.

### Post-irradiation thermal desorption from adsorbed (η^3^-C_3_H_5_)Ru(CO)_3_Cl

**Figure 3:**
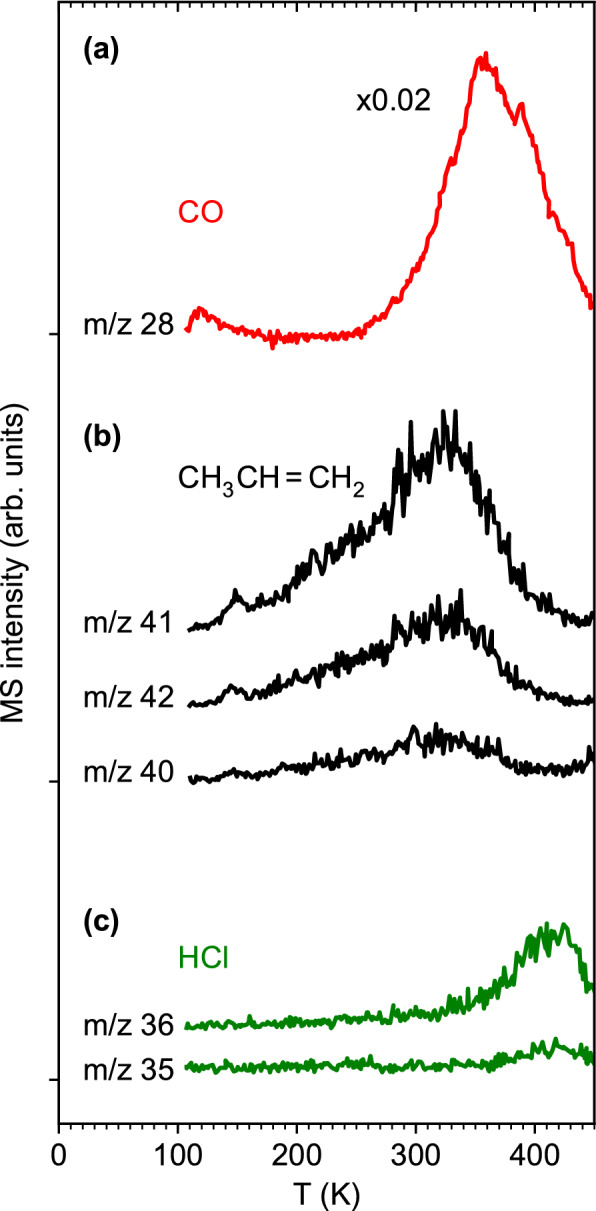
Thermal desorption spectra (TDS) acquired from an adsorbed layer of (η^3^-C_3_H_5_)Ru(CO)_3_Cl on Ta following an electron exposure of 1.25·10^17^ e^−^/cm^2^ at *E*_0_ = 31 eV. The selected *m/z* values give evidence of desorption of **(a)** CO, **(b)** propene, and **(c)** HCl.

Following the complete decay of the CO signal in ESD from the freshly prepared precursor layer, a post-irradiation TDS experiment was performed on each sample (see Fig. 1c). Figure 3 shows a representative result. Here, *m/z* 41 gives evidence that (η^3^-C_3_H_5_)Ru(CO)_3_Cl has in fact been mainly decomposed as the characteristic desorption peak near 220 K of the intact precursor (Supplementary Information, Fig. S2) is absent after electron exposure. In contrast, a broad signal with onset near 150 K and maximum around 300 K is present in the *m/z* 41 TDS curve after irradiation. Desorption signals with the same shape and maximum are seen in the *m/z* 40 and *m/z* 42 data. The relative intensities of these three signals agree well with the mass spectrum of propene (*m/z* 42 (70%), 41 (100%), 40 (30%)^19^). Physisorbed propene desorbs well below 100 K^21^ so that it should have evaporated at least partially if it had formed during electron irradiation which proceeded at 110 K. The fact that the observed product propene desorbs at a much higher temperature according to TDS indicates that it is formed not as an immediate consequence of electron irradiation but that a thermally activated reaction step must be involved.

Desorption of HCl with onset above 300 K and maximum above 400 K is evident from TDS data recorded at *m/z* 36 and *m/z* 35 and supported by the mass spectrum of HCl (*m/z* 36 (100%), 35 (18%)^19^). Similar desorption temperatures, i.e., a signal between 300 and 350 K, have been observed for HCl on Pt(111) at the lowest coverage^22^, and desorption of HCl from a single crystal alumina surface started around 300 K and peaked just below 400 K^23^. However, physisorbed HCl would again desorb below 100 K^22^. Consequently, the absence of signals at *m/z* 35 and *m/z* 36 in ESD (Fig. 2) supports that thermal activation is required to release HCl to the gas phase.

Finally, an intense signal with onset near 300 K and maximum around 360 K is present in the TDS curve for *m/z* 28. It is assigned to desorption of additional CO as confirmed by a mass spectrum acquired near 360 K in a separate experiment and revealing the absence of other products such as hydrocarbons or CO_2_ (*m/z* 44) (Supplementary Information, Fig. S3). The high temperature supports the release of CO through a thermal surface reaction.

Based on tabulated total ionization cross sections for CO, propene, and HCl^24,25^, and the fragmentation pattern of the mass spectra^19^, the partial ionization cross sections for the formation of CO^+^ from CO, C_3_H_5_^+^ from propene, and HCl^+^ from HCl can be derived as σ_BEB_(CO, *m/z* 28) = 2.35 Å^2^, σ_BEB_(propene, *m/z* 41) = 2.21 Å^2^, and σ_BEB_(HCl, *m/z* 36) = 3.53 Å^2^. The intensities observed for CO at *m/z* 28 and propene at *m/z* 41 in the present TDS experiments thus reflect approximately the relative amounts of these two products. Accordingly, propene seen in post-irradiation TDS amounts to roughly 2% of the quantity of CO released through thermal processing. Together with the even smaller hydrocarbon desorption signals during ESD as compared to CO (Fig. 2a), our results indicate that most of the allyl carbon is retained in the deposit. This information was obscured in previous XPS results by the strong overlap of the C 1 s and Ru 3d signals^14^ but evidence for a large amount of residual carbon was seen in the nanogranular structure of FEBID deposits fabricated from (η^3^-C_3_H_5_)Ru(CO)_3_Br^15^. The even smaller desorption signal at *m/z* 36 together with the larger partial ionization cross section for the parent ion of HCl further indicates that the quantity of HCl that desorbs during annealing (Fig. 3) following electron exposure of 1.25·10^17^ e^−^/cm^2^ (Fig. 2) is even smaller (< 1%). This estimate is in line with the previous result that most of the Cl content was retained on the surface after a similar electron exposure (8·10^16^ e^−^/cm^2^) of (η^3^-C_3_H_5_)Ru(CO)_3_Cl at 105 K and 500 eV^14^.

The relative amounts of CO released during electron irradiation and subsequent thermal desorption has been evaluated from the areas under the ESD and TDS curves recorded for *m/z* 28. As a result, the amount of CO that desorbs during electron exposure at 110 K exceeds the amount observed in TDS by a factor of roughly nine. This is close to the previously reported CO signal in XPS that decayed to roughly 20% of the initial intensity after a similar electron exposure (8·10^16^ e^−^/cm^2^) at 500 eV^14^, pointing again to the surprising efficiency of CO removal at 31 eV.

### Loss of CO monitored by reflection–absorption infrared spectroscopy

**Figure 4:**
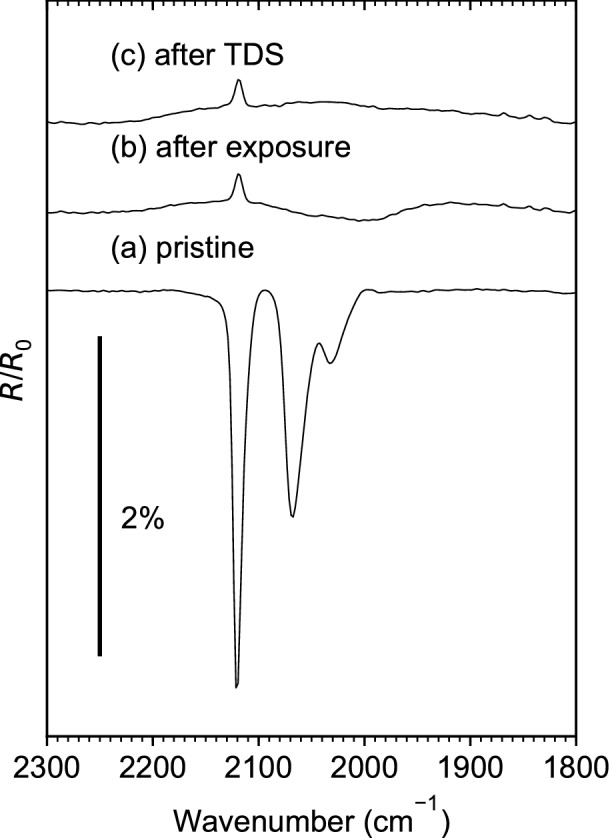
**(a)** Reflection absorption infrared spectrum (RAIRS) acquired on a pristine adsorbed layer of (η^3^-C_3_H_5_)Ru(CO)_3_Cl on Ta. **(b)** RAIRS of the same layer after an electron exposure of 1.25·10^17^ e^−^/cm^2^ at *E*_0_ = 31 eV. **(c)** RAIRS of the same layer after annealing to 450 K during a subsequent TDS experiment and final cooling to 110 K.

To monitor CO that remains on the surface even after thermal treatment, RAIRS experiments were performed on a pristine layer of (η^3^-C_3_H_5_)Ru(CO)_3_Cl, the same layer after the ESD experiment, and after subsequent TDS (Fig. 4), i.e., before and after the experimental steps visualized by Fig. 1b and c. The pristine layer (Fig. 4a) shows three intense CO stretching bands in line with a previous ATR-IR result^14^ but somewhat shifted to 2,122 cm^−1^, 2,072 cm^−1^, and 2,034 cm^−1^. These bands have disappeared after the ESD experiment leaving behind a broad band around 1990 cm^−1^ (Fig. 4b). This band coincides with the band positions of CO on Ru(0001) at low coverage^26^. Considering that the loss of CO during electron exposure proceeded simultaneously with reduction of the Ru center from Ru(II) to a state closer to metallic Ru^14^, we tentatively assign the broad band around 1990 cm^−1^ to CO that remains attached to such reduced metal sites. This signal has, however, disappeared after the TDS experiment (Fig. 4c) indicating that the thermal treatment, in fact, removed all residual CO.

### Electron-stimulated desorption from adsorbed (η^3^-C_3_H_5_)Ru(CO)_3_Cl in presence of NH_3_

**Figure 5:**
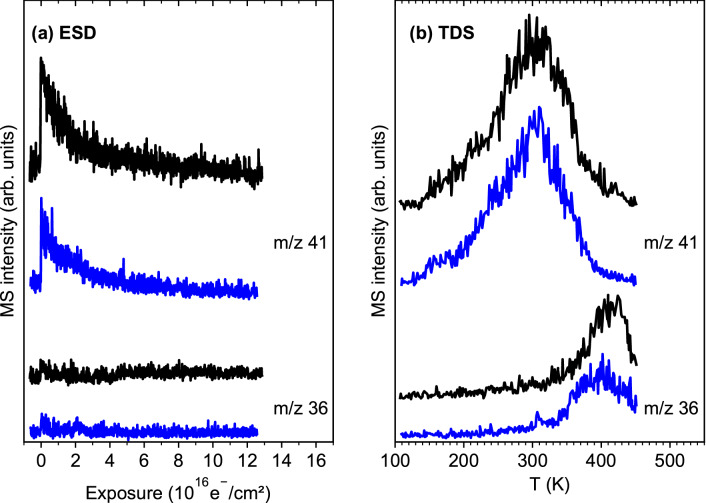
**(a)** Electron-stimulated desorption (ESD) signals at *m/z* 41 (C_3_ hydrocarbon) and *m/z* 36 (HCl) recorded during an electron exposure of 1.25·10^17^ e^−^/cm^2^ at *E*_0_ = 31 eV of (η^3^-C_3_H_5_)Ru(CO)_3_Cl on Ta held at 110 K. **(b)** Thermal desorption spectra (TDS) recorded following the same ESD experiments. The data plotted in black refer to an experiment performed in the absence of NH_3_ while the precursor layer was covered by NH_3_ in the case of the data plotted in blue.

In a control experiment, NH_3_ was condensed on top of a pristine layer of (η^3^-C_3_H_5_)Ru(CO)_3_Cl to assess if its presence enhances the removal of Cl from the precursor. Again, a combined ESD and TDS experiment was performed under the same conditions as described in the previous section. Figure 5 shows that the desorption signals for *m/z* 41 (C_3_ hydrocarbons) and *m/z* 36 (HCl) obtained with and without NH_3_ are very similar. In particular, an enhancement of the HCl production in presence of NH_3_ is clearly not observed.

We note that the temperature in the present experiments was close to the multilayer desorption temperature of NH_3_ as observed in experiments performed at lower temperature^21^ (see also “Methods”). Therefore, a TDS experiment was performed without prior electron exposure to verify that some NH_3_ actually sticks on the preadsorbed precursor layer. In fact, a desorption signal of NH_3_ set in sharply at the start of the temperature ramp and leveled off slowly to extend up to the desorption temperature of the precursor supporting adsorption of NH_3_ (Supplementary Information, Fig. S4). Consequently, the absence of enhanced HCl production (Fig. 5) demonstrates that NH_3_ does not efficiently react with (η^3^-C_3_H_5_)Ru(CO)_3_Cl upon electron exposure.

### Deposit purification by electron irradiation in presence of NH_3_ monitored by Auger electron spectroscopy

**Figure 6:**
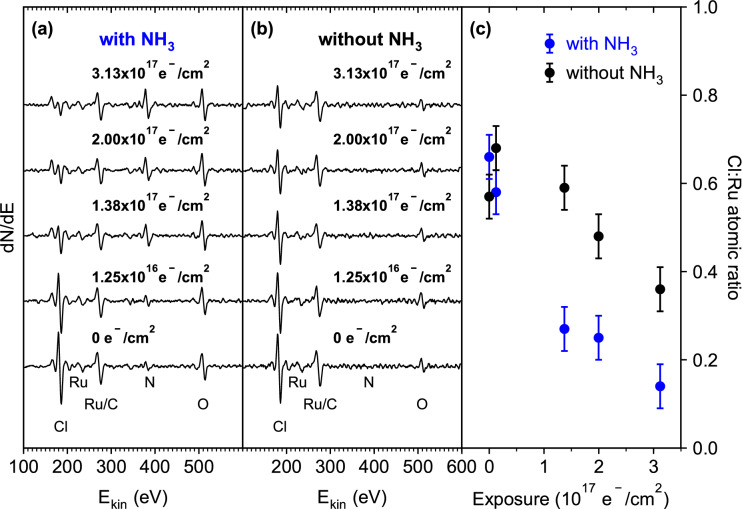
Auger electron spectra (AES) recorded on a deposit prepared by electron exposure (1.25·10^17^ e^−^/cm^2^) at *E*_0_ = 31 eV and subsequent annealing to 450 K of an adsorbed layer of (η^3^-C_3_H_5_)Ru(CO)_3_Cl on Ta before (bottom) and after an increasing number of purification cycles (from bottom to top) **(a)** in the presence of NH_3_ and **(b)** without NH_3_. Each purification cycle comprised an electron exposure of 1.25·10^16^ e^−^/cm^2^ followed by annealing to 450 K. **(c)** Relative amounts of Cl and Ru after increasing numbers of purification cycles performed on the deposit in presence of NH_3_ (blue) and on the deposit only (black). These values were determined from the peak-to-peak intensities of the Cl signal at 184 eV and the Ru signal at 235 eV by accounting for the respective sensitivity factors for 5 keV impinging electrons [0.6941 for Ru (235 eV) and 8.1285 for Cl (184 eV)^28^].

In the set of experiments aiming at the deposit purification process, we investigated the efficiency of NH_3_ with respect to removal of Cl from a deposit prepared from (η^3^-C_3_H_5_)Ru(CO)_3_Cl by the sequence of ESD and TDS experiments described above (see Fig. 1b and c). A total of 25 purification cycles were performed on the deposit. In each cycle, NH_3_ was condensed on the deposit at 110 K (see Fig. 1d) and an electron exposure of 1.25·10^16^ e^−^/cm^2^ was applied at 31 eV during which ESD data were recorded (see Fig. 1e), followed by annealing to 450 K during a further TDS run (see Fig. 1f). AES was performed with the sample held at room temperature prior to the first cycle and after selected purification cycles (Fig. 6a). For comparison, the same sequence of experiments was performed without condensing NH_3_ on the deposit (Fig. 6b). It is obvious that the Cl content decreases more rapidly in the presence of NH_3_. This was quantified by evaluating the intensity of the Cl and Ru signals. Unfortunately, the more sensitive Ru Auger signal at 277 eV coincides closely with the C signal at 275 eV and can therefore not be used. Therefore, the relative intensities of the Cl signal (184 eV) and the smaller Ru signal at 235 eV have been determined from the AES data for purification experiments with and without NH_3_ (Fig. 6c). We note that the ratio Cl:Ru was roughly 0.6 already prior to the first purification cycle. Considering that only minor desorption of Cl was detected during deposit formation, a ratio near one would have been expected. However, a precise quantitative analysis of AES data requires careful evaluation of effects from background corrections, instrumental resolution, backscattered electrons, and layer structure of the sample^27,28^. Such a precise analysis is beyond the scope of the present work.

Note that some loss of Cl is also expected under the 5 keV AES electron beam^14^. Therefore, each AES was acquired on a new spot of the sample. The systematic decrease of the Cl signal with increasing number of purification cycles (Fig. 6a,c) thus results from the combined effects of electron irradiation at 31 eV in the presence of NH_3_ and the subsequent annealing step. In consequence, Fig. 6c clearly supports that the electron-induced removal of Cl from a deposit produced from (η^3^-C_3_H_5_)Ru(CO)_3_Cl is enhanced by the presence of NH_3_.

The AES data (Fig. 6a,b) also reveal the presence of nitrogen (389 eV) and oxygen (510 eV). In particular, the amount of surface-bound N keeps increasing with the number of purification cycles giving evidence of a reaction between NH_3_ and the surface. The AES signal of N was also observed when the purification cycles were applied to the clean Ta substrate (Supplementary Information, Fig. S5), indicating that N becomes chemically bonded to the underlying substrate. However, chemisorption of atomic N on Ru surfaces can occur^29,30^ so that a reaction with small Ru aggregates that emerge during purification of the deposit is also conceivable. The O Auger signal in Fig. 5 and Fig. S5 can be traced back to residual H_2_O in the vacuum chamber that reacts with the Ta surface (Supplementary Information, Fig. S6) and possibly also with the emerging metallic Ru^31^. Note that the different intensity of O in Fig. 5a and b relates to the time between the sputtering process and the purification experiment. This was longer in Fig. 5a where sputtering and purification were performed on different days. However, the amount of H_2_O accumulated during the time span of deposit fabrication is very small as compared to NH_3_ applied during the purification cycles (Supplementary Information, Fig. S7) Therefore, the effect of H_2_O on the deposit purification chemistry can be neglected.

### Electron-stimulated desorption during deposit purification in presence of NH_3_

**Figure 7:**
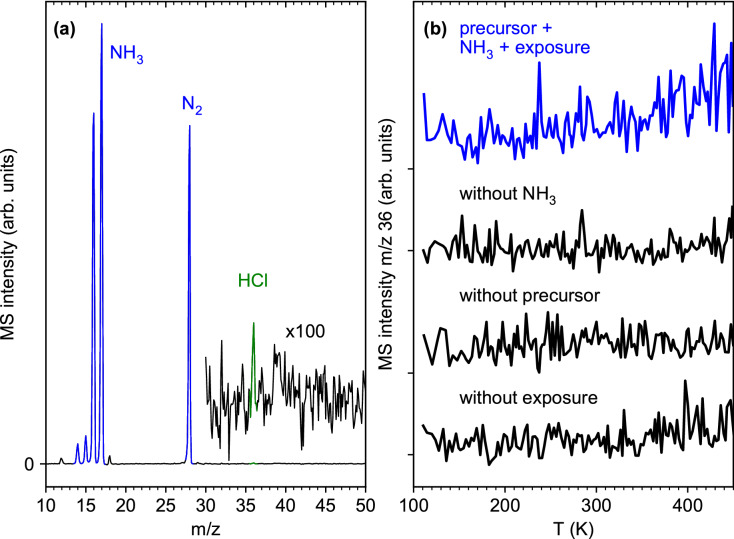
**(a)** Accumulated mass spectrum acquired during an electron exposure of 1.25·10^16^ e^−^/cm^2^ at *E*_0_ = 31 eV of the first deposit purification cycle in the presence of NH_3_ at 110 K as shown in Fig. 5a. Note a very small signal at *m/z* 36 that points to desorption of small amounts of HCl. **(b)** Thermal desorption spectra (TDS) acquired at *m/z* 36 following this electron irradiation as well as control experiments performed either without NH_3_, without prior electron exposure, and also without a precursor layer. Desorption of HCl occurs only when electron irradiation of the deposit is performed in the presence of NH_3_.

Unfortunately, the amounts of Cl and HCl are difficult to assess in a quantitative manner from the present ESD and TDS results, which raises questions about the fate of Cl during the purification steps. While ESD of HCl from cisplatin (*cis*-Pt(NH_3_)_2_Cl_2_) was clearly seen at *E*_0_ = 500 eV and room temperature^16^, electron irradiation during the present purification cycles was performed at 110 K and thus much below the desorption temperature of HCl (Fig. 2). However, accumulated mass scans performed during electron irradiation within the first purification cycle of Fig. 6a show a tiny signal of HCl at *m/z* 36 beside the strong desorption of NH_3_ and of its decomposition product N_2_ (Fig. 7a, experiment visualized in Fig. 1e). Furthermore, the TDS experiment performed at *m/z* 36 after the electron exposure of 1.25·10^16^ e^-^/cm^2^ also shows a small desorption signal with onset at 350 K (Fig. 7b, experiment visualized in Fig. 1e), despite the fact that this exposure was ten times smaller than that applied for deposit formation. This signal has not reached its maximum at 450 K, suggesting that more HCl must have desorbed during the subsequent bakeout period at 450 K (see “Methods”).

We note that NH_4_Cl was identified by RAIRS as an intermediate product of the electron-induced decomposition of cisplatin^16^. NH_4_Cl decomposes thermally only above 450 K^32^ and might, in consequence, trap Cl until further electron exposure at 5 keV during AES. Therefore, RAIRS was performed in a separate experiment before the first purification cycle, after electron exposure, and after annealing during TDS (Supplementary Information, Fig. S8). However, the signal of NH_4_Cl was absent so that formation of such a less volatile product is not responsible for the relatively low apparent yield of HCl during ESD and TDS.

## Discussion

The aim of this study was to assess the potential of NH_3_ to assist in the removal of Cl during electron-induced deposit formation from a thin adsorbate of (η^3^-C_3_H_5_)Ru(CO)_3_Cl or in a post-deposition purification process. AES shows that the presence of NH_3_ in fact enhances the removal of Cl from a model deposit (Fig. 6). After a total exposure of 3.1·10^17^ e^-^/cm^2^ at 31 eV in the presence of coadsorbed NH_3_ with intermittent annealing to 450 K, the Cl content has been reduced by roughly 75%. The same procedure performed without NH_3_ leads to a smaller reduction of approximately 40%.

We compare the observed loss of Cl to previous results for layers of (η^3^-C_3_H_5_)Ru(CO)_3_Br and (η^3^-C_3_H_5_)Ru(CO)_3_Cl at 105 K and with similar thickness as used here but for exposure at 500 eV^14^. Both compounds decompose with a very similar rate under electron exposure. For (η^3^-C_3_H_5_)Ru(CO)_3_Cl, longer exposures were not reported but the halide content was reduced by less than 10% after an exposure of 7.58·10^16^ e^−^/cm^2^ in close agreement to (η^3^-C_3_H_5_)Ru(CO)_3_Br^14^. The bromide complex can therefore also serve as reference. In particular, the Br content was reduced by roughly 35% after an electron exposure of 5.63·10^17^ e^−^/cm^2^^14^. This is close to the reduction of the Cl content by the present purification cycles in the absence of NH_3_. Note here that thermal processing can contribute to removal of Cl in our experiment so that the result does not allow us to directly compare the efficiency of the underlying electron-induced chemistry at 31 eV to that reported for 500 eV^14^. However, halide removal was obviously slower in the previous purification attempts by electron irradiation alone^14^ than in our present NH_3_-based process.

The previous study also explored the room temperature purification of a deposit produced at the same temperature in a FEBID-type process from (η^3^-C_3_H_5_)Ru(CO)_3_Cl^14^. AES revealed that the Cl content dropped to about 25% of its initial value during a post-deposition electron exposure of 7.9·10^18^ e^−^/cm^2^ at 3 keV. This is again slower than in the present NH_3_-based process where an electron exposure that was more than an order of magnitude smaller has achieved a similar effect. A more detailed comparison between these two experiments is difficult because the previous study did not report the thickness of the deposit^14^. However, we estimate that the electron beam fully penetrated the deposit because the signal of the underlying Ag substrate was still visible.

This enhancing effect of NH_3_ is similar to the case of cisplatin (*cis*-Pt(NH_3_)_2_Cl_2_) studied previously^16,17^. In particular, electron exposure removed Cl more rapidly from cisplatin than from the analogous precursor *cis*-Pt(CO)_2_Cl_2_, that was studied earlier under similar conditions^33^. It was proposed^16^ that electron impact ionization of NH_3_ triggers proton transfer to Cl^−^ (Eq. 1).

1 $${\text{NH}}_{{3}}^{ \cdot + } \, + \,{\text{Cl}}^{ - } \to {\text{HCl}}\, + \,{\text{NH}}_{{2}}^{ \cdot }$$

Also, electron-induced fragmentation yielding NH_x_ (x < 3) leads to release of atomic hydrogen (AH) that can react with the Cl ligand (Eq. 2).

2 $${\text{H}}^{ \cdot } \, + \,{\text{Pt}} - {\text{Cl}} \to {\text{HCl}}\, + \,{\text{Pt}}$$

Both reactions (1) and (2) yield HCl as observed in ESD from cisplatin^16^. Note again that the desorption efficiency of HCl was presumably higher for cisplatin than for (η^3^-C_3_H_5_)Ru(CO)_3_Cl studied here because ESD was performed at room temperature and higher *E*_0_. However, the electron-induced decomposition of cisplatin was very slow and ESD of HCl not visible when *E*_0_ was decreased to 50 eV. This underlines again that the close similarity between the decomposition rate of (η^3^-C_3_H_5_)Ru(CO)_3_Cl at 500 eV as reported before^14^ and at 31 eV as studied herein is unexpected and should be studied in more detail.

Fundamental insight into the electron-induced fragmentation of (η^3^-C_3_H_5_)Ru(CO)_3_X precursors via dissociative electron attachment (DEA) and dissociative ionization (DI) may point to an explanation for the unexpectedly high electron-induced decomposition rate of (η^3^-C_3_H_5_)Ru(CO)_3_Cl at 31 eV. In the case of (η^3^-C_3_H_5_)Ru(CO)_3_Br, DI leads to more comprehensive loss of ligands than DEA^13^. If DI is equally dominant for the Cl analogue, the cross section of the electron-induced fragmentation at 31 eV can be comparable or even higher than at 500 eV. This is suggested by comparison with the electron energy dependence of DI from Co(CO)_3_NO^34^. If, on the other hand, DEA was the dominant process, the larger yield of SEs at 500 eV should enhance the reactions as compared to lower *E*_0_. This latter kind of reactivity was held responsible for the much faster decomposition of cisplatin at 500 eV as compared to 50 eV^16^. However, absolute cross sections for DEA and DI would be needed to further support this interpretation.

Our attempts to enhance the electron-induced release of HCl by adsorbing NH_3_ on the pristine layer of (η^3^-C_3_H_5_)Ru(CO)_3_Cl prior to exposure were not successful. A possible reason is that NH_3_ interacts only weakly with the intact precursor layer. In fact, the present experiments were performed close to the multilayer desorption temperature of NH_3_^19^. Also, ESD of NH_3_ is already efficient at 35 K and 15 eV^19^ and obvious in the present experiments from the mass spectrum acquired during electron irradiation of the first deposit purification cycle (Fig. 7a), and from the nearly quantitative loss of NH_3_ vibrational bands in RAIRS after this irradiation (Supplementary Information, Fig. S8). In contrast to cisplatin where NH_3_ is directly bonded to the central metal atom in direct vicinity to the Cl ligands, the crowded coordination sphere of intact (η^3^-C_3_H_5_)Ru(CO)_3_Cl probably shields the Cl ligand from the weakly physisorbed NH_3_. In consequence, physisorbed NH_3_ molecules do not contribute noticeably to the decomposition of (η^3^-C_3_H_5_)Ru(CO)_3_Cl and removal of Cl. In contrast, the deposit produced by electron irradiation is depleted of CO so that coordination sites on the Ru center are more easily accessible to NH_3_, enabling a close approach to the Cl ligand. We thus propose that NH_3_ can coordinate to the Ru center of the precursor after some CO ligands have been removed by electron irradiation. This enhances the probability that electron irradiation ionizes or fragments NH_3_ near the Cl ligand which can consequently induce the formation of HCl, most likely in an intramolecular reaction.

The combination of ESD experiments at cryogenic temperature with TDS allow us to identify potential contributions of thermal reactions to the decomposition of (η^3^-C_3_H_5_)Ru(CO)_3_Cl under FEBID-type conditions, where the precursor is irradiated at or above room temperature^10^. In fact, the ESD and TDS results (Figs. 2, 3) show that roughly 10% of the CO ligands remain within the deposit at cryogenic temperature after irradiation but are removed by increasing the temperature to 450 K. Also, small amounts of C_3_ hydrocarbons, presumably formed through electron-induced reactions involving H transfer between two allyl ligands, desorb during electron exposure (Fig. 2). Additional propene and HCl desorb during the temperature increase (Fig. 3). However, our rough estimate of the quantities of these products demonstrates that these reactions are of minor relevance. This rationalizes why most of the carbon content and all of the Br ligands remain in the deposit when FEBID is performed with (η^3^-C_3_H_5_)Ru(CO)_3_Br^14,15^. Additionally, we note that desorption of C_3_ hydrocarbons was not seen during deposit purification (Fig. 7a). We therefore conclude that NH_3_ is a suitable reagent to enhance the removal of Cl and possibly of other halides but is not beneficial with respect to removal of carbon.

## Conclusion

NH_3_ present during post-deposition electron irradiation enhances the removal of Cl from deposits produced by electron-induced decomposition of (η^3^-C_3_H_5_)Ru(CO)_3_Cl. Intramolecular reactions of the Cl ligands with NH_3_ adsorbed on coordination sites of Ru that were liberated by electron-induced and thermal loss of CO are held responsible for this enhancement. In contrast, thermal reactions contribute to the desorption of CO but remove only minor amounts of the allyl and Cl ligands.

## Methods

### Precursor synthesis

Synthesis was carried out under an inert atmosphere (N_2_) using standard Schlenk techniques. Reagents were purchased from Acros Organics, Oakwood Chemical, and Fisher Scientific and used without further purification. ^1^H NMR spectra (Supplementary Information, Fig. S9) were obtained on a 400 MHz Bruker spectrometer and signals were referenced to the residual protons of CDCl_3_. IR Spectroscopy (Supplementary Information, Fig. S10) was performed on a Perkin Elmer Spectrum One Fourier transform infrared spectrometer using a solution cell equipped with NaCl windows and a path length of 1.0 mm. Synthesis and purification of (η^3^-C_3_H_5_)Ru(CO)_3_Cl was carried out using a literature procedure^35^. The compound was characterized by comparison to literature data^35^. 1H NMR (300 MHz, CDCl_3_) δ 5.29 (tt, 1H, J = 8.7, 13.2 Hz), 4.20 (dd, 2H, J = 8.7, 1.0 Hz), 2.98 (dd, 2H, J = 13.2, 1.0 Hz). IR (heptane) 2,111, 2,062, 2,016 cm^−1^.

The integrity of the compound after shipping to Bremen was checked up to a sublimation temperature of 200 °C by EI-MS which shows the parent ion as well as the characteristic series of ligand losses (Supplementary Information, Fig. S11).

### UHV setup

All experiments were performed in an ultrahigh vacuum (UHV) setup described previously^10, 36^ with a base pressure of about 10^–10^ mbar. It contains a polycrystalline Ta sheet held at 110 K by liquid N_2_ cooling. The sample temperature is controlled by resistive heating of two thin Ta ribbons spot-welded to the thicker Ta sheet and is measured using a type E thermocouple press-fitted to the Ta substrate. The setup is equipped with a quadrupole mass spectrometer (QMS) residual gas analyser (Stanford, 300 amu) with electron impact ionization at 70 eV, a commercial flood gun (SPECS FG 15/40) for electron irradiation, an Auger electron spectrometer (STAIB DESA 100), and a sputter gun operated with Ar^+^ ions. All Auger electron spectra (AES) were recorded using an electron energy of 5 keV.

### Preparation of adsorbed layers and estimate of thickness

Sample preparation was performed in line with previously reported procedures^10^. Prior to an experiment, the substrate was sputter-cleaned using Ar^+^ ions at 3 keV until the AES signals of the underlying Ta were clearly visible and any other signals, in particular, remaining Ru and C signals had disappeared. Immediately before each precursor deposition, adsorbed volatile compounds from the residual gas were further removed by annealing to 450 K through resistive heating of two thin Ta ribbons spot-welded to the thicker Ta sheet. The precursor (η^3^-C_3_H_5_)Ru(CO)_3_Cl was condensed on the Ta sheet at 110 K. This was done by introducing the precursor via a gas handling manifold consisting of precision leak valves and a small calibrated volume where the absolute pressure is measured with a capacitance manometer. For each film deposition, a calibrated amount of vapour was leaked via a stainless steel capillary opening onto the Ta substrate. However, due to slow decomposition of the precursor within the reservoir, this vapour contained some CO and a hydrocarbon species deriving from the allyl ligand. Therefore, a pumping cycle was applied to the reservoir prior to each dosing of the precursor. Also, the substrate was heated to a temperature of 170 K after each introduction of vapour to remove any free ligands from the layer of (η^3^-C_3_H_5_)Ru(CO)_3_Cl which starts to desorb at a temperature around 200 K.

The desorption temperature of (η^3^-C_3_H_5_)Ru(CO)_3_Cl as well as the film thickness were estimated by thermal desorption spectrometry (TDS) performed after introducing varying amounts of vapour. The QMS was used to monitor desorbing species during application of a temperature ramp of 1 K/s to the sample. The data recorded at *m/z* 41 (Supplementary Information, Fig. S2) show a weak desorption signal with maximum around 240 K which rapidly saturates when the pressure drop in the manifold was increased to 2 mTorr and is therefore ascribed to the monolayer. A second peak with maximum at 220 K starts to increase upon saturation of the monolayer peak, i.e., when larger pressure drops were noted in the manifold, and is hence attributed to the successive layers no longer in contact with the substrate. All further experiments were performed on precursor layers produced by leaking an amount of vapour corresponding to a pressure drop of 5 mTorr in the manifold. According to our estimate, this yielded a 2–3 monolayer adsorbate of the precursor. Assuming that the molecular size of (η^3^-C_3_H_5_)Ru(CO)_3_Cl is roughly comparable to that of MeCpPtMe_3_ for which an effective diameter of 0.96 nm has been deduced^37^, 2–3 monolayers would result in an average thickness of 2–3 nm, similar to the thickness used previously^14^. However, it was noted from TDS data acquired from pristine precursor layers prior to the experiments reported herein that an additional desorption signal at higher temperature was occasionally present (Supplementary Information, Fig. S4). Considering the observed release of ligands and, in particular, CO from the precursor in the reservoir, this signal is most likely ascribed to an unknown volatile decomposition product. Therefore, the absolute amount of (η^3^-C_3_H_5_)Ru(CO)_3_Cl in the adsorbed layers varied somewhat with time.

For purification experiments, NH_3_ was leaked onto the substrate held at 110 K. Again, TDS performed after leaking varying amounts of NH_3_ vapour onto the Ta substrate revealed that the surface coverage increased with increasing pressure drop in the manifold (Supplementary Information, Fig. S12). As 110 K is close to the multilayer desorption temperature of NH_3_^21^, the desorption signals increase sharply at the onset of the temperature ramp. Therefore, the TDS data for NH_3_ do not allow us to safely identify the transition from monolayer to multilayer regime. NH_3_ adsorbates in all further experiments were prepared by leaking an amount of vapour corresponding to a pressure drop of 5 mTorr in the manifold. We assume that consequently, during each experiment, NH_3_ was present on the substrate with a coverage at least within the monolayer regime.

### Electron-induced degradation of (η^3^-C_3_H_5_)Ru(CO)_3_Cl

The electron-induced degradation of the (η^3^-C_3_H_5_)Ru(CO)_3_Cl layers was studied by electron-stimulated desorption (ESD) isothermal experiments as well as by subsequent TDS in line with a methodology described previously^10^. For ESD, the sample was kept at 110 K and exposed to electron irradiation from the flood gun. This electron source delivers electrons with tuneable kinetic energy (*E*_0_) at an estimated resolution of the order of 0.5–1 eV. Here, *E*_0_ was set to 31 eV in all experiments, resulting in currents as measured at the substrate (*I*_p_) of the order of up to 150 µA for an irradiated area of 5 cm^2^. All ESD data were corrected by the background spectrum of residual gases. In the case that a mass spectrum was acquired during ESD, the background mass spectrum of the UHV chamber as measured immediately before starting the irradiation was subtracted. In experiments that monitored specific *m/z* ratios during electron exposure, the intensity levels before the start of irradiation and after its end were used to define a linear baseline to be subtracted.

After each electron irradiation, a TDS experiment was performed by applying a temperature ramp up to 450 K with a heating rate of 1 K/s to monitor products that desorb thermally from the degraded precursor layer. Subsequently, the sample was held at 450 K for typically 30 s to remove further volatile substances (bakeout). In each TDS, the signals of up to four selected characteristic masses were recorded. To evaluate the effect of NH_3_ on the electron-induced decomposition of the precursor, NH_3_ was dosed onto a layer of (η^3^-C_3_H_5_)Ru(CO)_3_Cl. After this preparation, ESD and subsequent TDS were again performed.

### Purification experiments

For purification experiments, a deposit was produced by performing electron irradiation on an adsorbed layer of (η^3^-C_3_H_5_)Ru(CO)_3_Cl followed by annealing to 450 K in a TDS experiment and subsequent bakeout. The cryostat was then allowed to warm up to room temperature for AES analysis. Subsequently, the substrate was again cooled to 110 K followed by condensation of NH_3_ on the deposit. The sample was then subjected to electron irradiation to an exposure of 2000 µC/cm^2^ (1.25·10^16^ e^−^/cm^2^) at *E*_0_ = 31 eV, followed by a further TDS run applying annealing to 450 K. This purification cycle was repeatedly applied. The elemental composition after selected purification cycles was monitored again by AES at room temperature. Each AES was acquired on a new spot of the sample to exclude contributions of the high-energy electron gun of the Auger spectrometer to the changes in composition. In a control experiment with the same sequence of cycles, the effect of irradiation in the absence of NH_3_ was studied.

In addition, electron-induced reactions of NH_3_ with the underlying Ta substrate in the absence of precursor were also monitored (Supplementary Information, Fig. S5). Finally, oxygen signals that showed up in the AES data were traced back to oxidation of the Ta substrate by residual H_2_O in the vacuum chamber (Supplementary Information, Fig. S6). However, comparison of TDS curves acquired from an NH_3_ adsorbate layer and on the clean Ta substrate after a waiting time corresponding to an entire ESD and TDS cycle revealed that the contribution of H_2_O to the electron-induced precursor decomposition must be negligible (Supplementary Information, Fig. S7).

## Supplementary information

Supplementary file1 (DOCX 1948 kb)

## Data Availability

The data sets generated during and/or analysed during the current study are available from the corresponding author upon reasonable request.
