# Supplementary material for: Efficient NH3-based process to remove chlorine from electron beam deposited ruthenium produced from (η3-C3H5)Ru(CO)3Cl
Source: Sci Rep. 2020 Jul 2;10:10901. doi: 10.1038/s41598-020-67803-y (PMC7331610; doi:10.1038/s41598-020-67803-y)
Supplement: Supplementary file 1 — Supplementary file1 (DOCX 1948 kb) [file 41598_2020_67803_MOESM1_ESM.docx]

Supplementary information to

Efficient NH_3_-based process to remove chlorine from electron beam deposited ruthenium produced from (η^3^-C_3_H_5_)Ru(CO)_3_Cl

Markus Rohdenburg*^1^, Hannah Boeckers^1^, Christopher R. Brewer^2^, Lisa McElwee-White^2^, and Petra Swiderek*^1^

Address: ^1^Institute for Applied and Physical Chemistry (IAPC), Fachbereich 2 (Chemie/Biologie), University of Bremen, Leobener Str. 5 (NW2), 28359 Bremen, Germany and ^2^Department of Chemistry, University of Florida, Gainesville, Florida 32611-7200, United States

Email: Markus Rohdenburg - m.rohdenburg@uni-bremen.de
and Petra Swiderek – swiderek@uni-bremen.de

* Corresponding authors


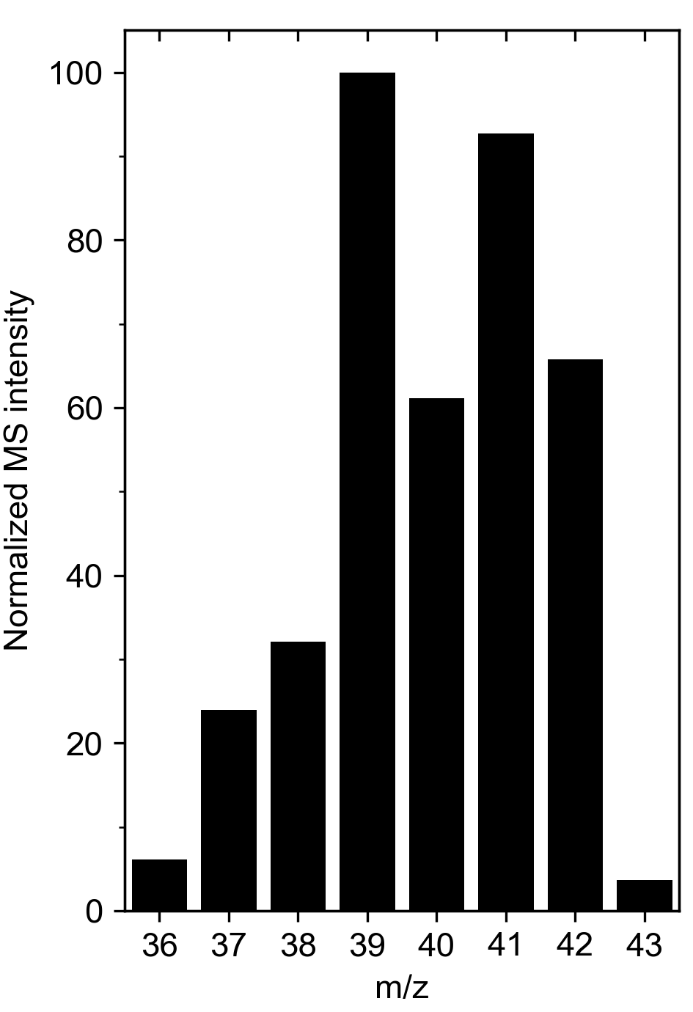


**Figure S1**: Weighted sum of the mass spectra of propene (H_2_C=CH-CH_3_) and propadiene (H_2_C=C=CH_2_) taken from Ref. 19 of main text. This composed spectrum roughly matches the ESD mass spectrum observed during the initial stage of electron exposure of an adsorbed layer of (η^3^-C_3_H_5_)Ru(CO)_3_Cl on Ta.


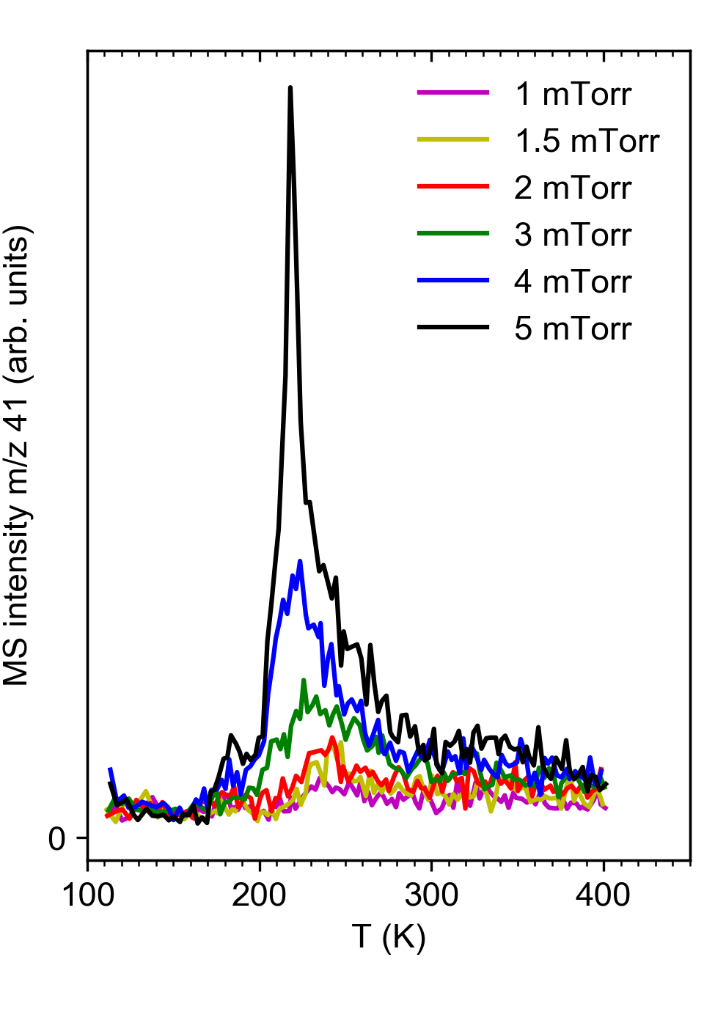


**Figure S2**: Thermal desorption spectra (TDS) of adsorbed (η^3^-C_3_H_5_)Ru(CO)_3_Cl acquired after leaking varying amounts of vapour from the manifold onto the Ta substrate held at 110 K. The weak desorption signal with maximum around 230 K saturates rapidly when the pressure drop in the manifold reaches 2 mTorr and is therefore ascribed to the monolayer coverage of the precursor. At higher amounts of vapour, a multilayer desorption signal develops with maximum at 210 K.


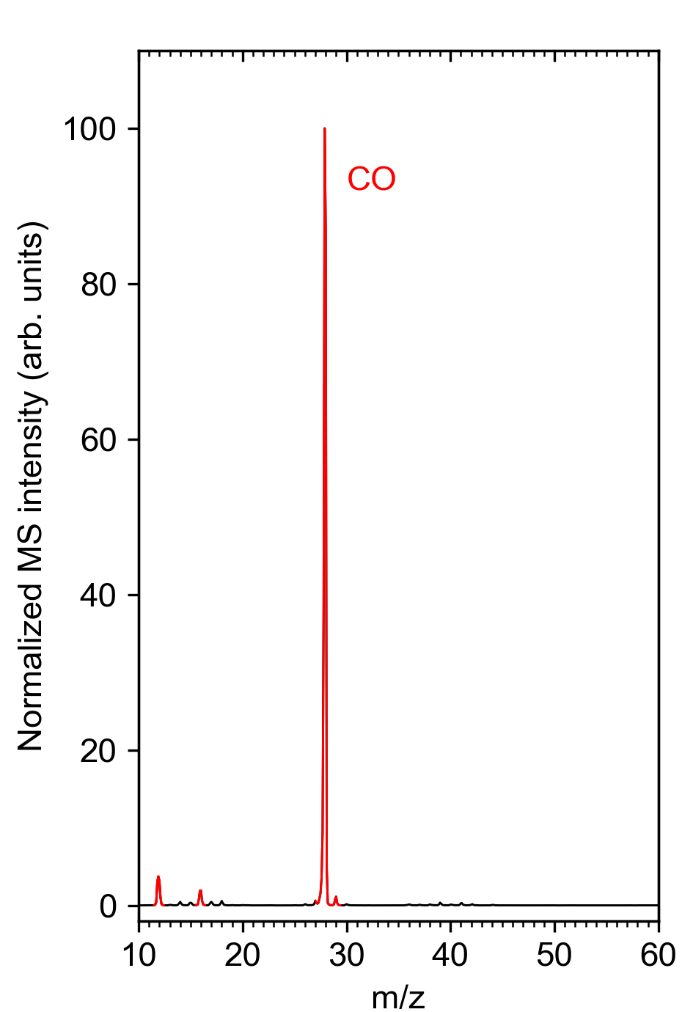


**Figure S3**: Mass spectrum recorded during a TDS run performed on an adsorbed layer of (η^3^-C_3_H_5_)Ru(CO)_3_Cl on Ta after an electron exposure of 1.25·10^17^ e^-^/cm^2^ at *E*_0_ = 31 eV. The data were acquired during temperature increase from 357 to 369 K and give evidence that the major desorption signal in the *m/z* 28 TDS curve shown in Fig. 2 is due to desorption of CO as supported by comparison with a literature mass spectrum of CO [19].


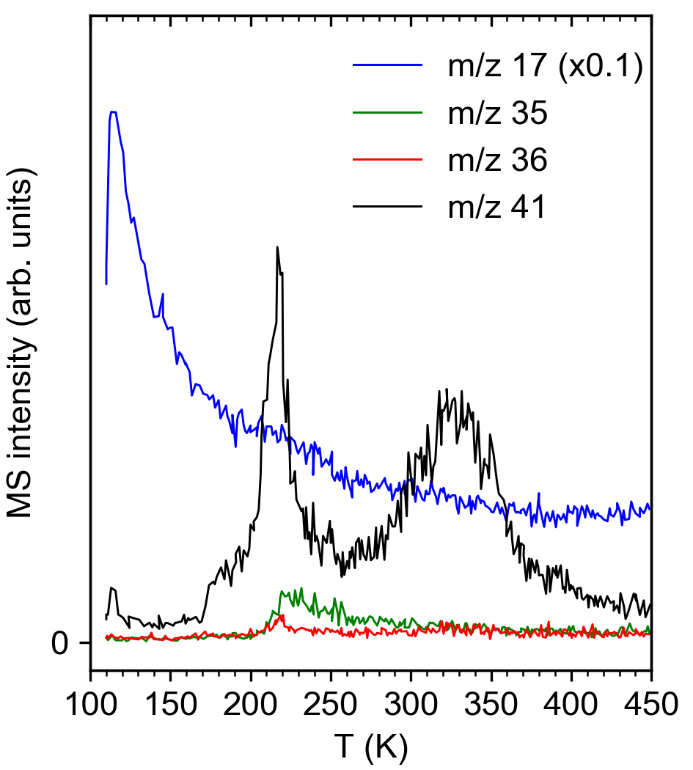


**Figure S4**: Thermal desorption spectra (TDS) of an adsorbed layer of (η^3^‑C_3_H_5_)Ru(CO)_3_Cl on Ta onto which NH_3_ has been adsorbed at 110 K. The *m/z* 17 desorption signal gives evidence that some NH_3_ sticks to the precursor layer under the given experimental conditions.


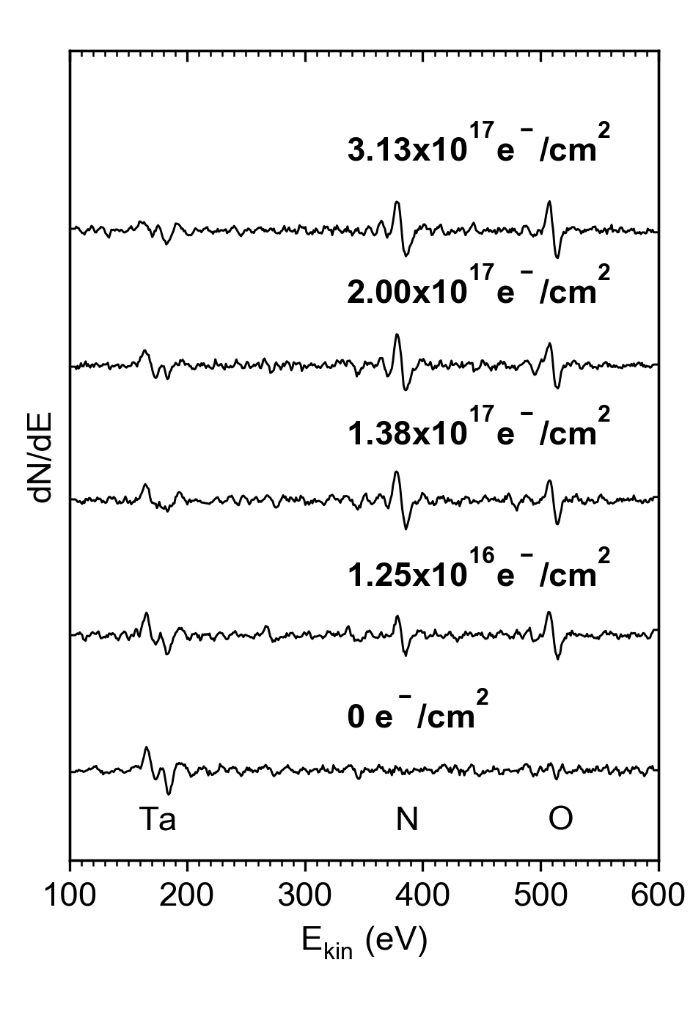


**Figure S5**: Auger electron spectra (AES) recorded on the freshly sputtered Ta substrate before (bottom) and after an increasing number of purification cycles (from bottom to top) in the presence of NH_3_. Each purification cycle comprised an electron exposure of 1.25·10^16^ e^-^/cm^2^ followed by annealing to 450 K. The increase of the nitrogen signal at 389 eV gives evidence that NH_3_ reacts with the Ta surface during the purification cycle. The increase of the oxygen signal at 510 eV is assigned to reactions with residual H_2_O present in the vacuum chamber.


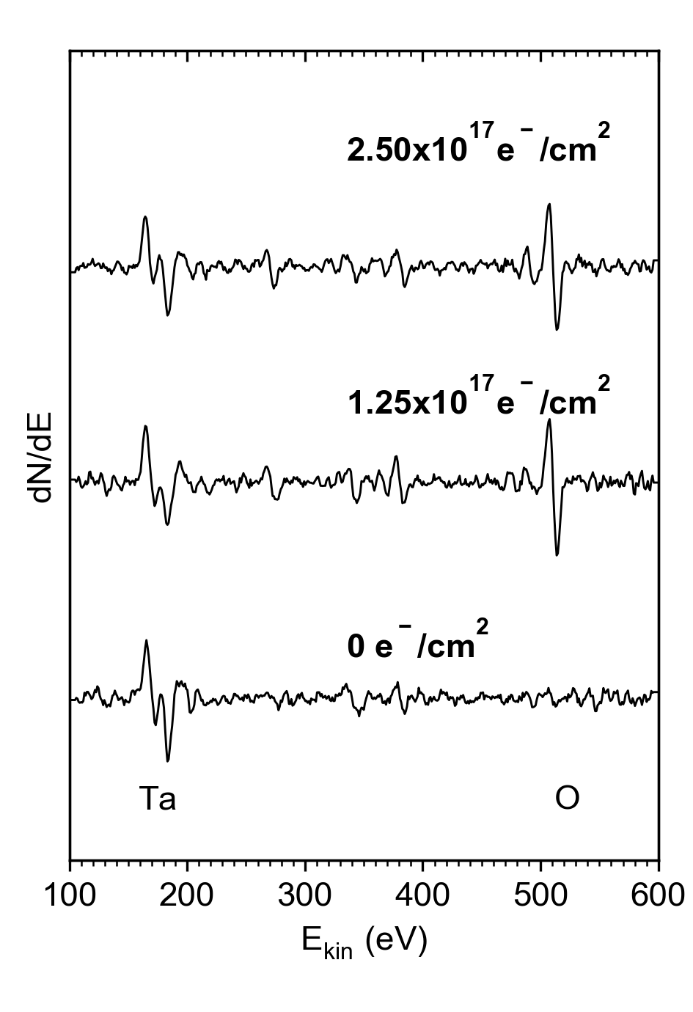


**Figure S6**: Auger electron spectra (AES) recorded on the freshly sputtered Ta substrate before (bottom) and after increasing electron exposure at *E*_0_ = 31 eV (from bottom to top). The increase of the oxygen signal at 510 eV is assigned to reactions of the Ta surface with residual H_2_O present in the vacuum chamber.


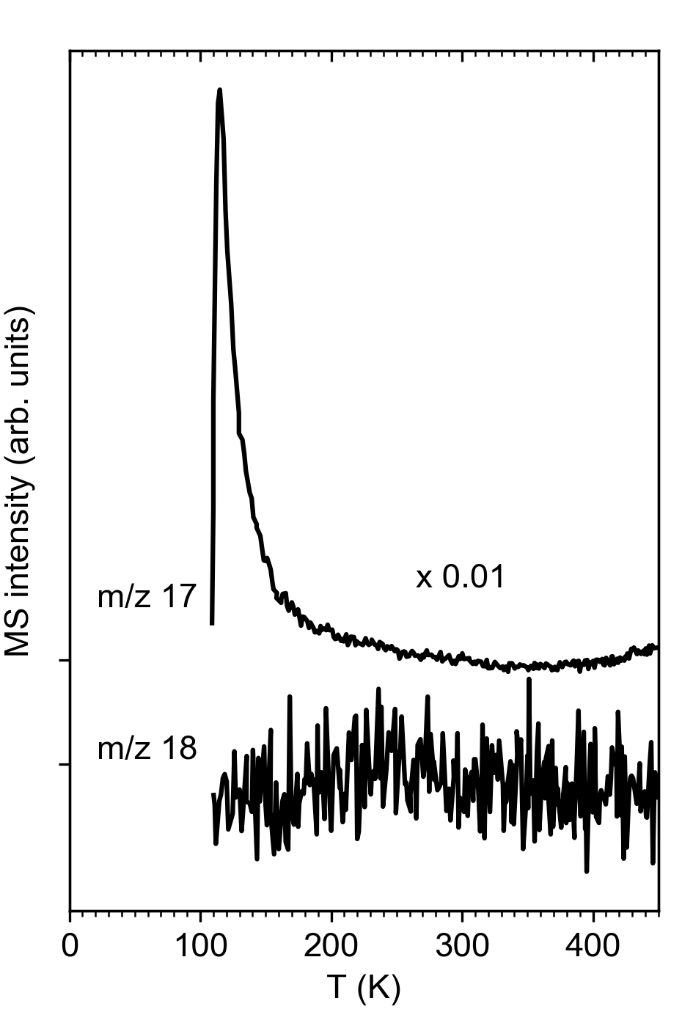


**Figure S7**: Thermal desorption spectrum (TDS) of an adsorbed layer of NH_3_ on Ta formed at 110 K from the same amount of vapour as applied in a single purification cycle (top). TDS curve recorded at *m/z* 18 after sputter cleaning of the Ta substrate and subsequent exposure of the substrate held at 110 K to the vacuum chamber residual gas for a time span corresponding to an entire deposit fabrication experiment as in Fig. 1b and Fig. 2 (bottom). The lack of obvious desorption signal in the *m/z* 18 curve demonstrates that the negligible amounts of physisorbed H_2_O accumulated during the time span of a deposit fabrication experiment are very small as compared to NH_3_ applied during a purification cycle.


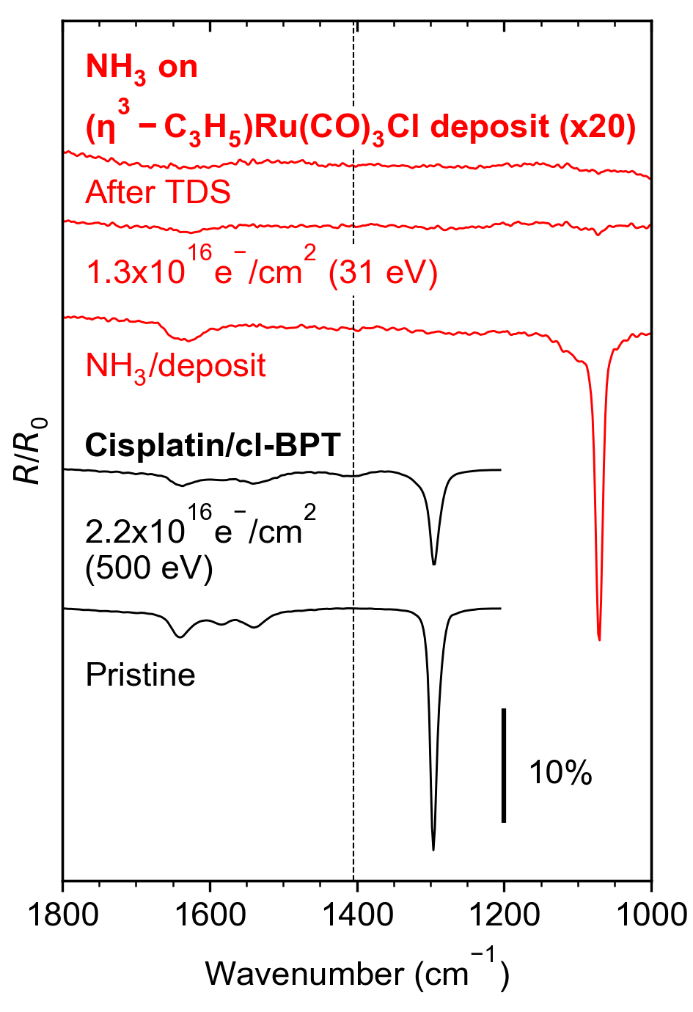


**Figure S8**: Reflection absorption infrared spectra (RAIRS) recorded following the initial deposit formation and dosing of NH_3_ (denoted as NH_3_/deposit), after an electron exposure of 1.25·10^16^ e^-^/cm^2^ at 31 eV and 110 K, and after the subsequent temperature increase to 450 K (top, plotted in red). Comparison to previous results for cisplatin recorded before and after electron exposure (bottom, plotted in black, data taken from [16]). The small signal at 1405 cm^-1^ present in irradiated cisplatin is ascribed to NH_4_Cl.


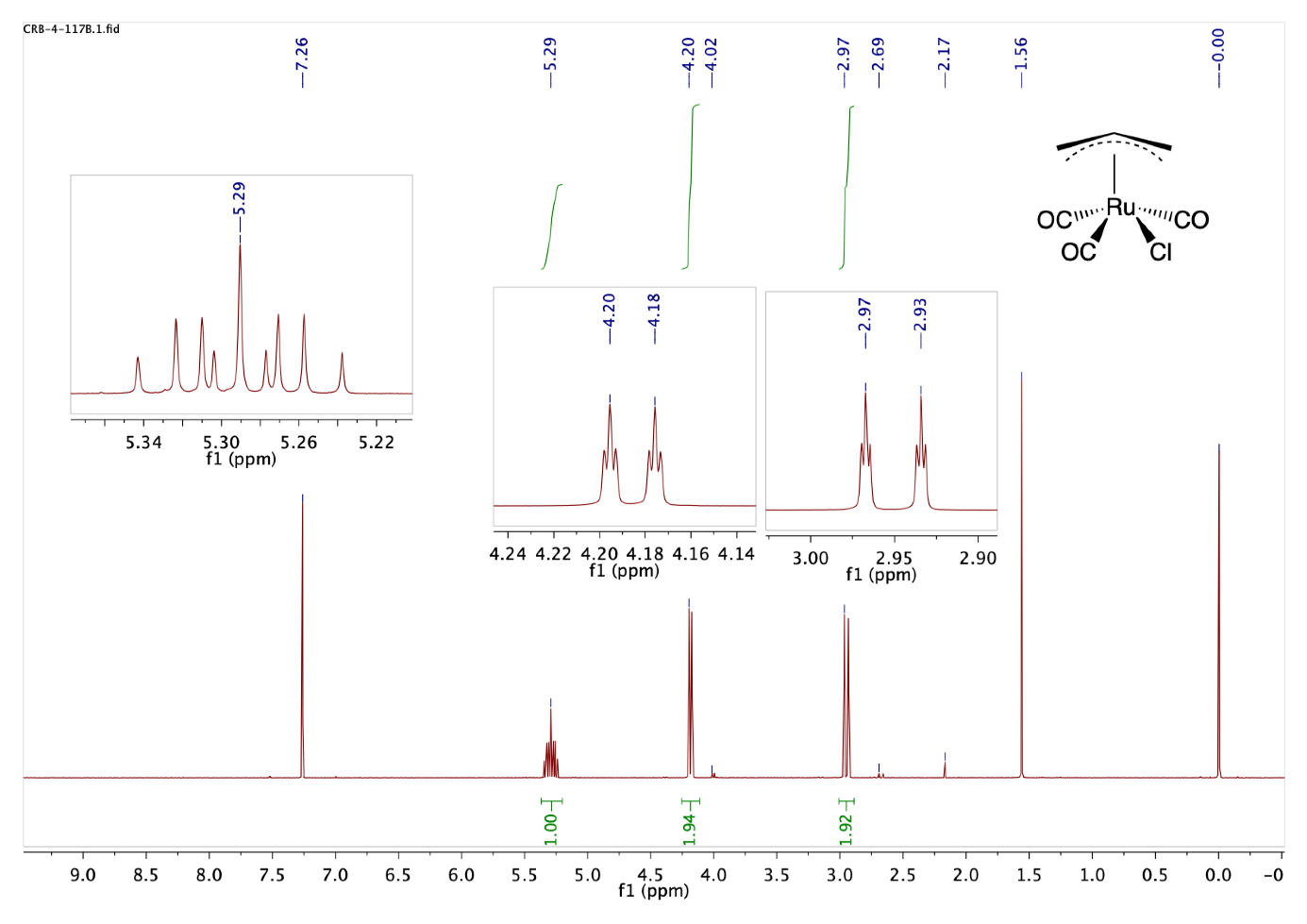


**Figure S9**: ^1^H NMR spectrum of (η^3^-C_3_H_5_)Ru(CO)_3_Cl in CDCl_3_.


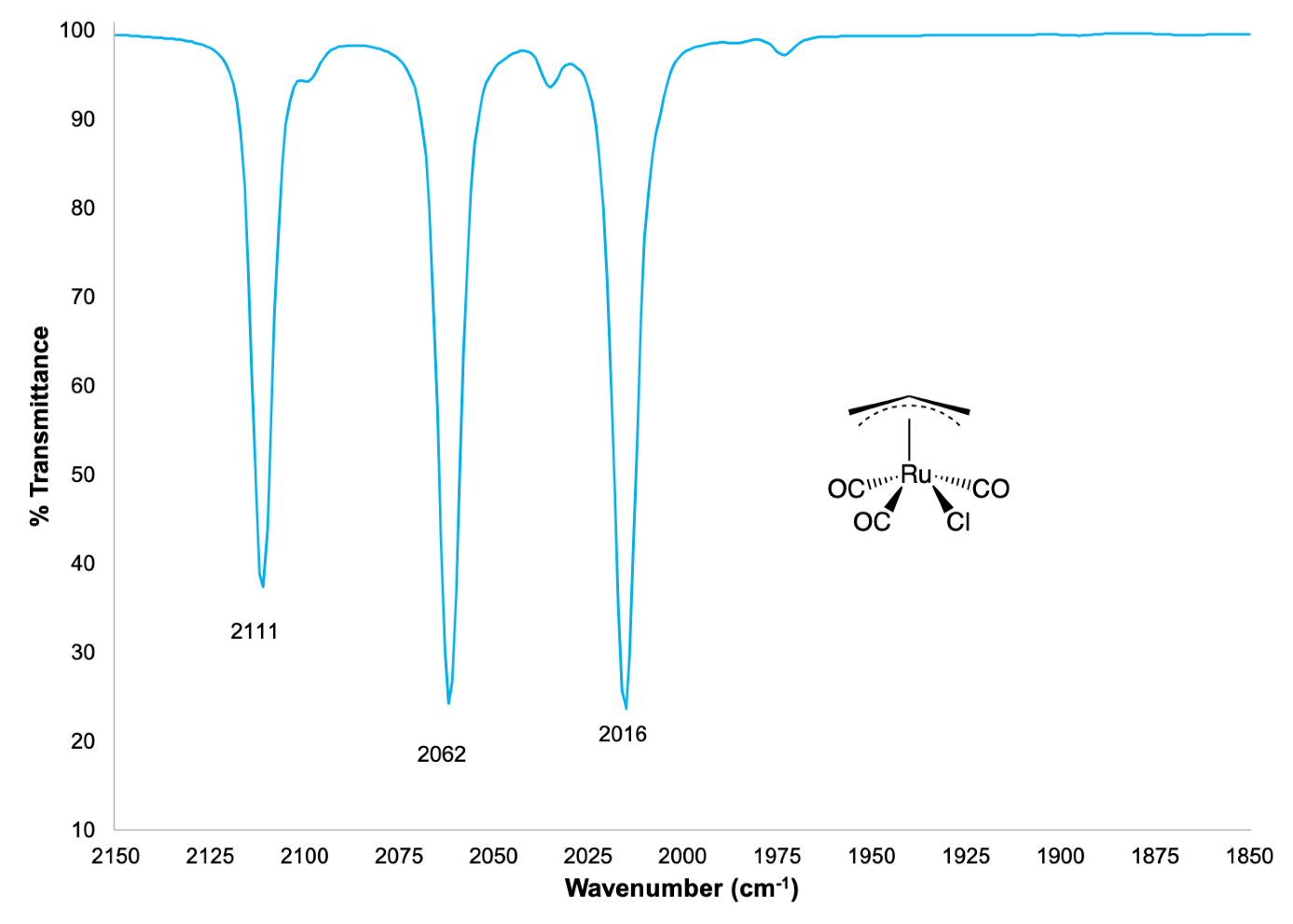


**Figure S10**: FTIR spectrum of (η^3^-C_3_H_5_)Ru(CO)_3_Cl in heptane.


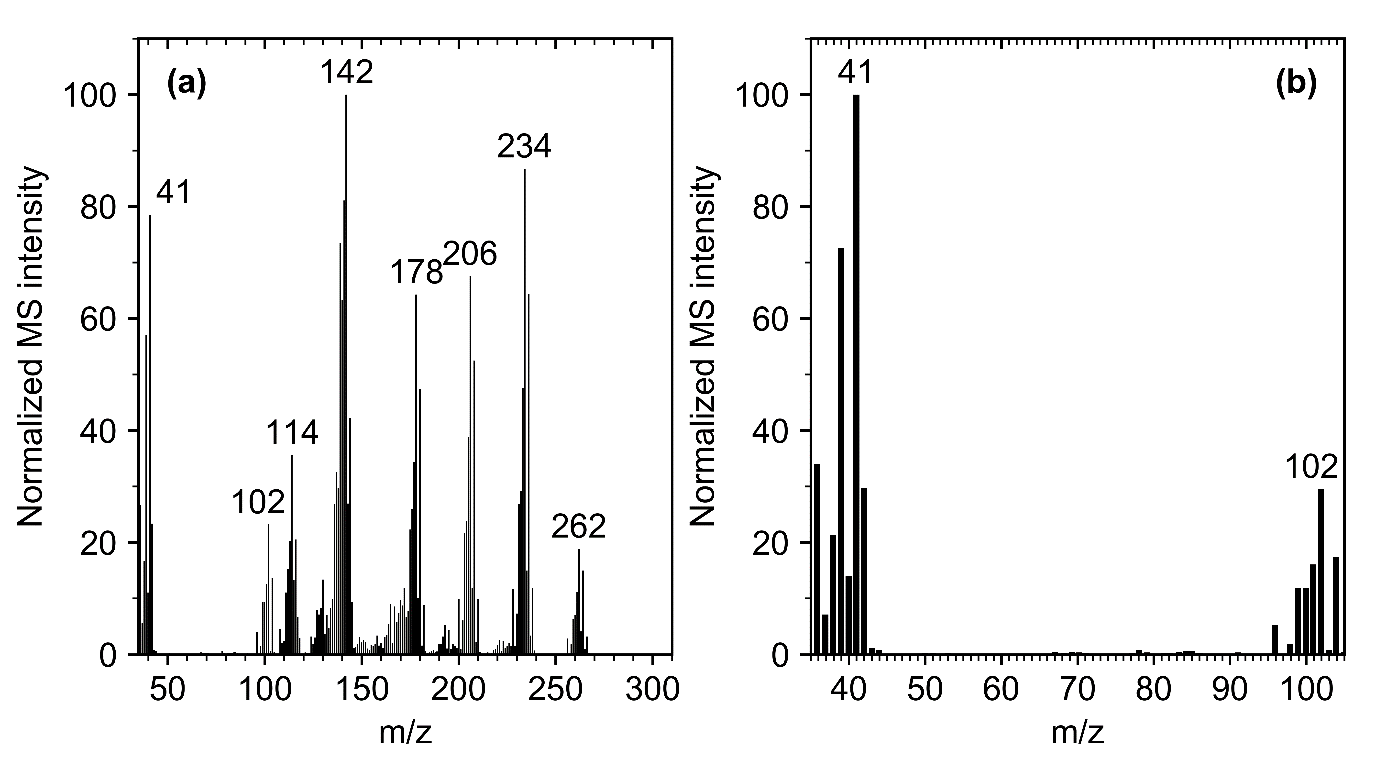
**Figure S11**: EI-MS of (η^3^-C_3_H_5_)Ru(CO)_3_Cl measured in Bremen after shipping of the compound.


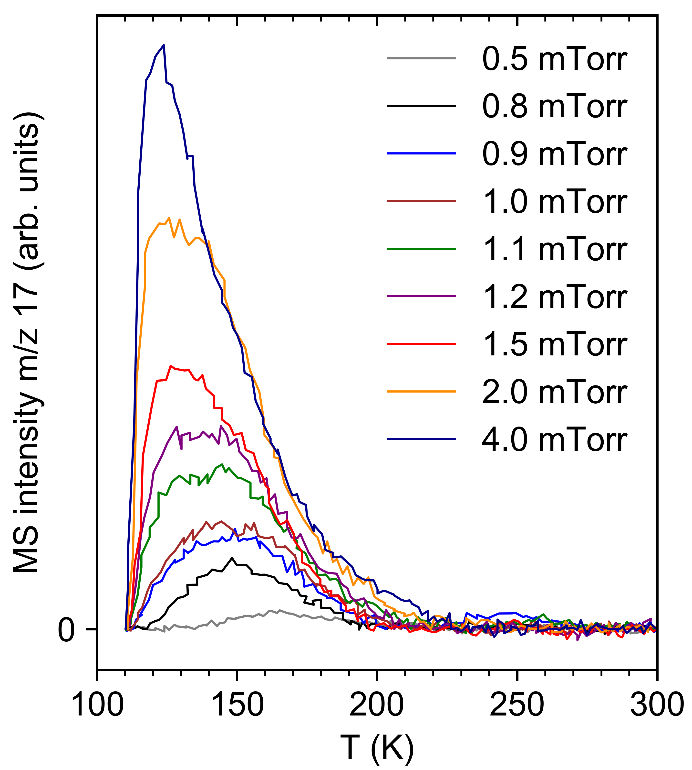


**Figure S12**: Thermal desorption spectra (TDS) of adsorbed NH_3_ acquired after leaking varying amounts of vapour from the manifold onto the Ta substrate held at 110 K.
